# Specific lipid magnetic sphere sorted CD146-positive bone marrow mesenchymal stem cells can better promote articular cartilage damage repair

**DOI:** 10.1186/s12891-024-07381-6

**Published:** 2024-04-01

**Authors:** Hanru Ren, Lele Zhang, Xu Zhang, Chengqing Yi, Lianghao Wu

**Affiliations:** Department of Orthopaedics, Shanghai Pudong Hospital, Fudan University, Pudong Medical Center, No. 2800, Gongwei Road, Shanghai, 200120 China

**Keywords:** Bone marrow mesenchymal stem cells, Lipid magnetic spheres, CD146, Cartilage damage, Sodium alginate

## Abstract

**Background:**

The characteristics and therapeutic potential of subtypes of bone marrow mesenchymal stem cells (BMSCs) are largely unknown. Also, the application of subpopulations of BMSCs in cartilage regeneration remains poorly characterized. The aim of this study was to explore the regenerative capacity of CD146-positive subpopulations of BMSCs for repairing cartilage defects.

**Methods:**

CD146-positive BMSCs (CD146 + BMSCs) were sorted by self-developed CD146-specific lipid magnetic spheres (CD146-LMS). Cell surface markers, viability, and proliferation were evaluated in vitro. CD146 + BMSCs were subjected to in vitro chondrogenic induction and evaluated for chondrogenic properties by detecting mRNA and protein expression. The role of the CD146 subpopulation of BMSCs in cartilage damage repair was assessed by injecting CD146 + BMSCs complexed with sodium alginate gel in the joints of a mouse cartilage defect model.

**Results:**

The prepared CD146-LMS had an average particle size of 193.7 ± 5.24 nm, an average potential of 41.9 ± 6.21 mv, and a saturation magnetization intensity of 27.2 Am^2^/kg, which showed good stability and low cytotoxicity. The sorted CD146 + BMSCs highly expressed stem cell and pericyte markers with good cellular activity and cellular value-added capacity. Cartilage markers Sox9, Collagen II, and Aggrecan were expressed at both protein and mRNA levels in CD146 + BMSCs cells after chondrogenic induction in vitro. In a mouse cartilage injury model, CD146 + BMSCs showed better function in promoting the repair of articular cartilage injury.

**Conclusion:**

The prepared CD146-LMS was able to sort out CD146 + BMSCs efficiently, and the sorted subpopulation of CD146 + BMSCs had good chondrogenic differentiation potential, which could efficiently promote the repair of articular cartilage injury, suggesting that the sorted CD146 + BMSCs subpopulation is a promising seed cell for cartilage tissue engineering.

**Supplementary Information:**

The online version contains supplementary material available at 10.1186/s12891-024-07381-6.

## Background

Cartilage tissue is a highly specialized connective tissue, due to the fact that articular cartilage lacks vascularity and innervation, contains only a single chondrocyte, and is surrounded by a large amount of extracellular matrix, resulting in poor self-repair and regeneration of cartilage tissue after injury [1, 2]. Once an injury occurs, it often leavespain or dysfunction, and may eventually develop into severe osteoarthritis [3]. Currently, the main clinical treatments for cartilage injuries such as pharmacological interventions, joint cavity cleaning, bone marrow stimulation techniques or microfracture are mainly for temporary symptomatic relief and do not regenerate healthy cartilage tissue [4–7]. In recent years, cartilage tissue engineering technology has brought new hope for regenerative repair of articular cartilage injury [8].

Cartilage tissue engineering mainly includes seed cells, cytokines and scaffold materials. Among them, seed cells are the key factor for the success of cartilage tissue engineering [9]. Mesenchymal stem cells (MSCs), with the advantages of strong proliferative capacity and multidirectional differentiation potential, have gradually become alternative seed cells for cartilage tissue engineering, with bone marrow mesenchymal stem cells (BMSCs) being the most common [10, 11]. BMSCs are currently the most widely used stem cells and have been shown to be able to differentiate into osteoblasts, chondrocytes and adipocytes [12–15]. In addition, MSCs are not composed of a single type of cell. Still, rather a heterogeneous population of cells that cannot be effectively identified and separated [16], in which different subpopulations of cells have different self-renewal and differentiation abilities, and this cellular heterogeneity of MSCs affects their therapeutic outcome [17, 18]. During the formation and development of human organs, MSCs originate from perivascular cells and outer membrane cells, and melanoma cell adhesion molecule (CD146) is one of the major molecular markers of pericytes [19]. CD146 is a transmembrane glycoprotein and a member of the immunoglobulin superfamily. In human bone marrow, CD146 is expressed in membranous reticulocytes, defined as perisinusoidal cells, which are capable of self-renewal after transplantation and lose their self-renewal capacity upon loss of CD146 expression [19, 20].

The study showed an increased in glycosaminoglycan/DNA content after enrichment of CD146-positive BMSCs [21]. The combination of CD146-positive adipose-derived mesenchymal stem cells with articular cartilage extracellular matrix scaffold can promote better cartilage regeneration in the long term [22]. CD146-positive human exfoliated deciduous teeth had higher osteogenic differentiation potential compared [23]. The CD146-positive subpopulation of human umbilical cord cells treats arthritis and provides an anti-inflammatory protective microenvironment by inhibiting interleukin 6 [24]. CD146-positive chondrogenic progenitor cells have higher levels of MSC-specific marker expression and better chondrogenic differentiation capacity [25]. Therefore, we hypothesized that the CD146-positive subpopulation of BMSCs has the potential to better promote cartilage repair. In this study, we successfully sorted out CD146-positive bone marrow mesenchymal stem cells (CD146 + BMSCs) by using an active target recognition method with the self-developed CD146-specific lipid magnetic spheres (CD146-LMS), compared the characteristics of BMSCs and CD146 + BMSCs, including morphology, gene expression, and chondrogenic differentiation ability, and determined their roles in the repair of articular cartilage injury with a view to laying a theoretical foundation for the use of CD146 + BMSCs in the repair of cartilage defects.

## Methods

### Materials

BMSCs cells were purchased from Beijing Beina Chuanglian Biotechnology Institute (China). DMEM medium, DMEM/F12 cell culture medium, and FBS were purchased from Gibco (United States of America). Fe_3_O_4_ solution, O-carboxymethyl chitosan octadecyl quaternary ammonium salt (OQCMC), dioleoyl Phosphatidylcholine (DOPC), and dimethyloctadecyl epoxypropylammonium chloride (GHDC) were purchased from Xi'an Kaixin Biotechnology Co (China). PEG-modified distearoyl phosphatidylethanolamine (DSPE-PEG) was purchased from Chongqing Yuyin Pharmaceutical Technology Co (China). Methylene chloride, cholesterol, N-hydroxysuccinimide (NHS) and 1-(3-Dimethylaminopropyl)-3-ethylcarbodiimide hydrochloride (EDC) were purchased from Shanghai Kether Chemical Technology Co (China). CD105, CD73, CD45, HLA-DR, recombinant sex-determining region y box protein 9 (Sox9), CD90, Aggrecan, Collagen II antibodies, CD34, CD166, and CD146 were purchased from Abcam (United Kingdom). BCA Protein Concentration Assay Kit was purchased from Beyotime (China). Hematoxylin Stain was purchased from Wuhan Xavier Biotechnology Co (China). Eosin stain was purchased from Zhuhai Beyotime Biotechnology Co (China).

### Preparation and experimental procedure of CD146-LMS

**Fig. 1:**
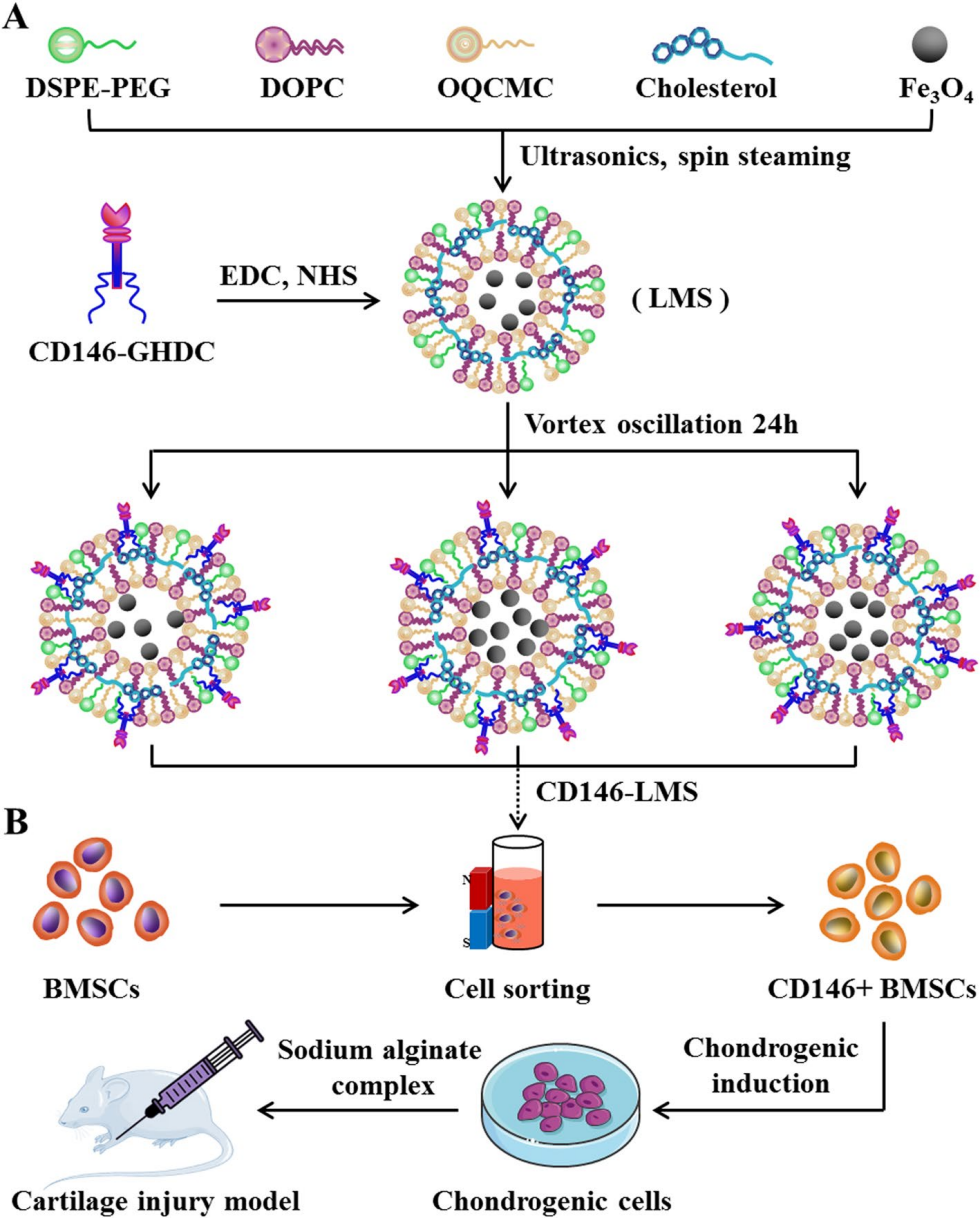
Preparation process and experimental flow chart of CD146-LMS. **A** Preparation process of CD146-LMS; (**B**) Flow of sorting, inducing differentiation and repairing cartilage defects in mice of BMSCs

In this study, for the first time, a BMSCs sorting scheme using CD146 lipid magnetic balls was designed (Fig. 1A). CD146-LMS was prepared by reverse evaporation method. 10 mg of cholesterol, 5 mg of DOPC, 5 mg of OQCMC, and 1 mg of DSPE-PEG were co-solubilized with dichloromethane as a solvent, then 10 mL of Fe_3_O_4_ (10 mg/mL) solution was added, and ultrasonication was performed with shaking for 6 min under the condition of ultrasonication power of 27%. The dichloromethane was removed after rotary evaporation for 30 min. LMS aqueous solution was obtained. Take 60 µg CD146 antibody dissolved in 1 mL GHDC isopropanol solution (1 mg/mL), add 0.2 mg NHS and 0.2 mg EDC respectively, and leave overnight at 4 °C. Add 1 mL CD146-GHDC solution into 1 mL LMS solution, vortex for 5 min, and then store it at 4 °C, and the continuous reaction for 24 h can obtain CD146-LMS. CD146-LMS was added to BMSCs cells to capture CD146 + BMSCs. CD146 + BMSCs cells induced differentiation and complexed with sodium alginate to form microspheres. The microspheres were injected into the joints of cartilage-deficient mice to assess the role of the CD146 subpopulation of bone marrow mesenchymal stem cells in cartilage damage repair (Fig. 1B).

### Characterization test

A BI-90 Plus laser particle sizer was used to test the particle size and potential of CD146-LMS. A UV spectrophotometer was used to scan the UV absorption spectrum of CD146-LMS. A magnetic property measurement system (MPMS) was used to test the magnetization intensity of CD146-LMS. Fourier transform infrared spectroscopy (FTIR) was used to test the infrared spectrum of CD146-LMS. X-ray diffraction (XRD) was used to test the crystalline properties of CD146-LMS. Raman spectra of CD146-LMS were tested using Raman spectroscopy. The morphology and distribution of CD146-LMS were observed by transmission electron microscopy (TEM), scanning electron microscopy (SEM), and atomic force microscopy (AFM).

### In vitro cytotoxicity and BrdU staining assay

BMSCs cells were added to 96-well plates with 1000 cells per well. 2.5, 5, 7.5, and 10 μM CD146-LMS were added to the cells and incubated in a 37 °C, 5% CO_2_ incubator for 24 h. Subsequently, 20 μL MTT was added to each well of cells, and 200 μL of DMSO was added after continuing the incubation for 2 h. Absorbance was measured at a wavelength of 560 nm using an enzyme meter for 5 days, and cell growth curves were recorded and plotted. BMSCs cells were co-cultured with 10 μM CD146-LMS for 3 d. The cells were incubated for 40 min after adding 10 μL BrdU, 15 min after adding 300 μL of 4% paraformaldehyde, 10 min after adding 300 μL of HCl solution, 5 min after adding 300 μL of HCl solution, and 10 min after adding 100 μL of boric acid solution. min, incubated for 20 min after adding 0.3% Triton solution, closed for 1 h after adding 200 μL of 4% goat serum, incubated overnight after adding 200 μL of BrdU primary antibody, incubated for 2 h after adding secondary antibody, stained with DAPI for 10 min, and observed and photographed under the microscope.

### Cell sorting and surface antigen detection

Purified CD146 + BMSCs were obtained by adding BMSCs cells in a 2 mL sterile eppendorf tube, incubating with 20 μL of CD146-LMS for 15 min, and then magnetically separating the cells for 10 min and then abandoning cell fluid. CD146 + BMSCs were washed by adding 1 mL of DMEM medium and transferred to 1% gelatin-coated 6-well plates. The magnetic beads were removed after placing them in the incubator for 48 h, and the cells were passaged when their growth density reached 80% to 90%. The morphology and growth of P2—P5 generation cells were observed by microscopy and hematoxylin–eosin (HE) staining. The expression of cell surface antigens HLA-DR, CD166, CD90, CD45, CD105, CD34, CD73, and CD146 in P3 generation CD146 + BMSCs cells was detected by flow assay.

### Chondrogenic induction of differentiation and properties test

P3 generation BMSCs and CD146 + BMSCs were digested with trypsin and counted, and 1 × 10^6^ cells were added into 15 mL sterile centrifuge tubes, centrifuged at 1200 r/min for 5 min, and added into a chondrogenic induction medium for induced differentiation. Experimental groups: (1) Ordinary medium group (NC); (2) Chondrogenic induction medium group, including BMSCs group and CD146 + BMSCs group (H-DMEM containing 10% FBS, 50 mg/mL streptomycin, 50 μg/mL vitamin C, 1% ITS, 40 μg/mL proline, 100 nmol/L dexamethasone, 50 U/mL penicillin and 10 ng/mL TGF-β1), and the liquid was changed once every 72 h. Inverted phase contrast microscopy was used to observe the characteristics of cell morphology changes. After 3 weeks of induction, chondrogenic properties were detected by alizarin red staining, type II collagen staining, and AB-PSA staining. The protein expression of Sox9, Collagen II, and Aggrecan was detected by Western blot. 12% polyacrylamide gel was prepared for electrophoresis, and after electrophoresis, the membrane was rotated at 350 mA for 2 h. After 2 h of closure, the membrane was incubated overnight with the primary antibody, and the secondary antibody was incubated for 2 h and then detected by a chemiluminescence system. The primary antibody was incubated overnight at 4 °C in the refrigerator, and the secondary antibody was incubated at 37 °C for 2 h. RT-PCR detected the expression of Collagen II, Sox9, and Aggrecan genes, and GAPDH was used as a control (Table 1). The results were analyzed using the 2^−∆∆Ct^ method.

**Table 1:** List of genes and their specific primer sequences for RT-PCR validation

Gene name	Primer	Sequence
Aggrecan	Forward primer	5'-GGACTTCCGCTGGTCAGATG-3'
	Reverse primer	5'-GTTTGTAGGTGGTGGCTGTG-3'
CollagenII	Forward primer	5'- CCGTGCTCCTGCCGTTTC-3'
	Reverse primer	5'- CTGAGGCAGTCTTTCACGTCT-3'
Sox9	Forward primer	5'- TCTGAACGAGAGCGAGAAGC-3'
	Reverse primer	5'- CCGTTCTTCACCGACTTCCT-3'
GAPDH	Forward primer	5'- AATGGGCAGCCGTTAGGAAA-3'
	Reverse primer	5'- GCGCCCAATACGACCAAATC-3'

### Animal model and sample detection

BMSCs and CD146 + BMSCs cells were induced for 3 weeks and prepared as a single cell suspension at a concentration of 5.0 × 10^7^/mL, and 1 mL was taken and mixed with 1.5% sodium alginate solution. Subsequently, sodium alginate microspheres encapsulating the cells were prepared by adding CaCl_2_ solution (102 mmol/L) to the mixture of cells and sodium alginate, and the above processes were carried out under aseptic conditions. The microspheres encapsulating the cells were placed in a petri dish and continued to be cultured for one week after the addition of a chondrogenic induction medium. The condition of the cells within the microspheres was observed under a microscope after staining with 0.01% acridine orange. Eighteen BALB/c female nude mice were divided into 3 groups of 6 mice each. They were anesthetized using 1% sodium pentobarbital and then sterilized. A 1.0 cm long curved incision was made at the patella of the hind limb of the mice, and part of the lateral femoral muscle and lateral joint capsule was incised to expose the articular surface of the distal femur. Small holes were drilled in the middle of the articular surface using a syringe needle with an external diameter of 1.2 mm to create a mouse articular cartilage injury model. The induced BMSCs and CD146 + BMSCs composite sodium alginate microspheres were filled in the small holes created by the needle. The control group was filled with sodium alginate microspheres without wrapped cells, the BMSCs group was filled with sodium alginate microspheres encapsulating BMSCs, and the CD146 + BMSCs group was filled with sodium alginate microspheres encapsulating CD146 + BMSCs. Finally, the wounds were rinsed using 0.9% saline the skin was sutured, and the mice were returned to their cages for rearing after they were awake [26–29]. After 4 weeks of normal feeding, the mice were executed, and the joints were removed and observed for repair. Paraffin sections were made after decalcification using a nitric acid solution. HE staining and toluidine blue staining were used to observe the tissue repair, and the tissue repair was scored according to the semi-quantitative scoring criteria for articular cartilage designed by Pineda et al. Lower scores indicate better repair (Table 2) [30–32].

**Table 2:** Histology pineda scoring scale

Observation indicators		Score
Cartilage composition	Normal	0
	Mostly hyaline cartilage	1
	Mixed hyaline and fibrocartilage	2
	Mostly fibrocartilage	3
	Non-chondrocyte or tissue-free filling	4
Cartilage matrix staining	Normal	0
	Slightly reduced	1
	Reduced	2
	Significantly reduced	3
	No staining	4
Flatness of cartilage surface	Flatness (> 3/4)	0
	Medium flatness (1/2 ~ 3/4)	1
	No flatness (1/4 ~ 1/2)	2
	Very no flatness (< 1/4)	3
Cartilage thickness	> 2/3	0
	1/3 ~ 2/3	1
	< 1/3	2
Integration of cartilage and peripheral cartilage	Both graft edges bonded	0
	One graft edge bonded	1
	Neither edge bonded	2
Subchondral bone morphology	Normal cancellous bone	0
	Some of the cancellous bone is hardened	1
	sclerotic bone	2
	Sclerotic bone and some fibrous tissue	3
	Fibrous tissue only or no tissue filling	4
Subchondral bone and peripheral osseous integration	Both graft edges bonded	0
	One graft edge bonded	1
	Neither edge bonded	2

### Statistical methods

Statistical significance was analyzed using SPSS 22.0, and groups were statistically significant at *P* < 0.05 by t-test or one-way ANOVA (*P < 0.05; **P < 0.01; ***P < 0.001).

## Results

### Characterization test

**Fig. 2:**
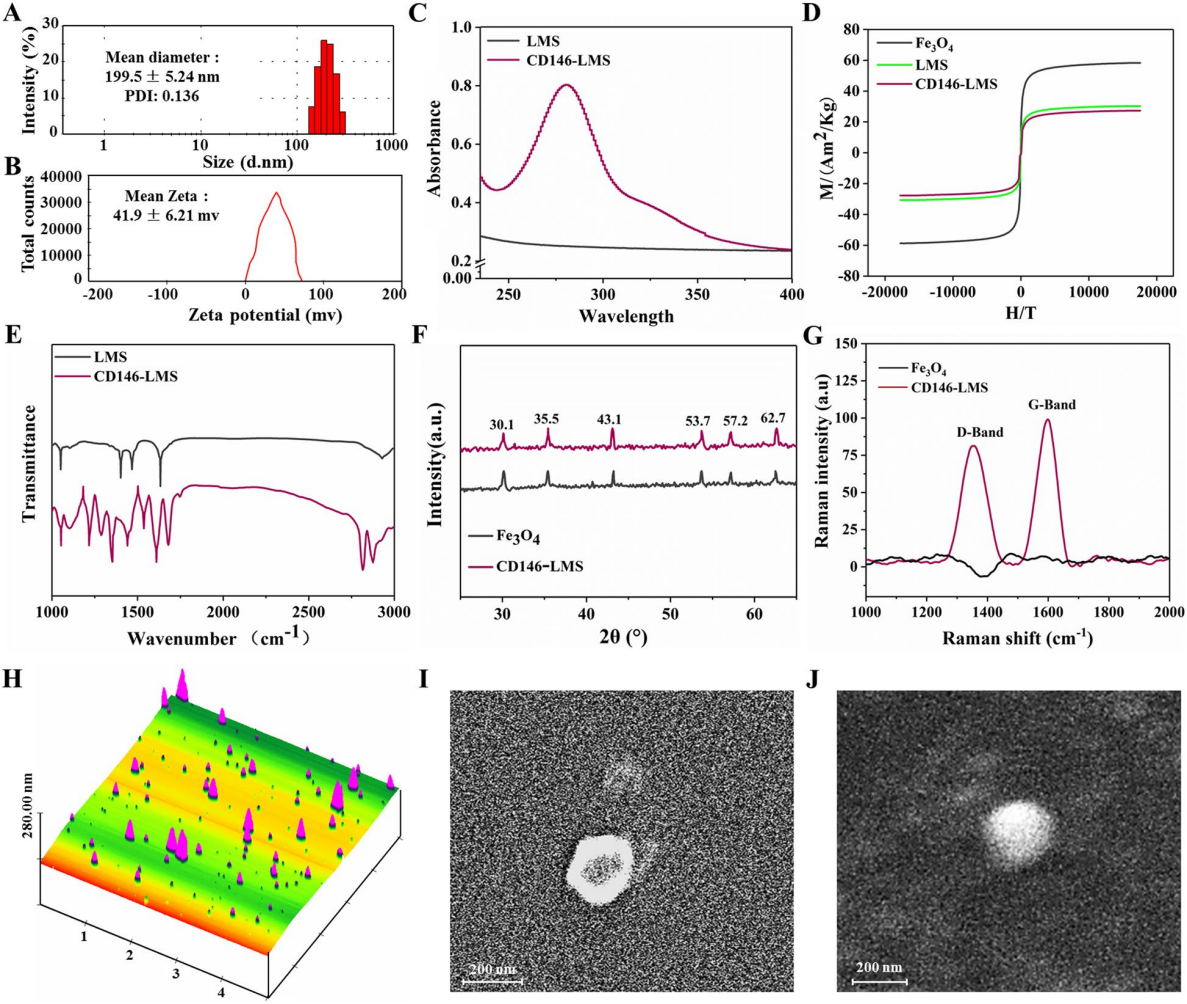
CD146-LMS characterization test. **A** Particle size test; (**B**) Potential test; (**C**) UV scanning pattern; (**D**) Hysteresis return curve; (**E**) Infrared scanning pattern; (**F**) Magnetic crystallization curve; (**G**) Raman spectrogram; (**H**) AEM observation; (**I**) SEM observation; (**J**) TEM observation

The average particle size of CD146-LMS was 199.5 ± 5.24 nm with a polydispersity index (PDI) of 0.136 (Fig. 2A). The charge of CD146-LMS was 41.9 ± 6.21 mv (Fig. 2B). LMS showed no absorption peak at 280 nm, and CD146-LMS showed the characteristic UV absorption peak of the protein at 280 nm, indicating the presence of CD146 antibody protein on the surface of the magnetic sphere (Fig. 2C). The saturation magnetization intensities of Fe_3_O_4_, LMS, and CD146-LMS were 58.2 Am^2^/Kg, 30.2 Am^2^/Kg, and 27.2 Am^2^/Kg, respectively, showing that the saturation magnetization intensity was weakened after liposomes encapsulated Fe_3_O_4_. There was no significant change in the saturation magnetization intensity of LMS coupled with CD146 antibody (Fig. 2D). FT-IR spectra showed that CD146-LMS showed new peaks at 2840—2930 cm^−1^, which were the characteristic absorption peaks of long carbon chains and methyl groups on quaternary ammonium salts, indicating the presence of GHDC on CD146-LMS, which was coupled to CD146, indirectly indicating that the CD146 antibody had been coupled to the surface of CD146-LMS (Fig. 2E). The XRD results of CD146-LMS showed six diffraction peaks (2θ = 30.1°, 35.5°, 43.1°, 53.7°, 57.2°, and 62.7°). These characteristic peaks corresponded to the crystalline types (220, 311, 400, 422, 511, and 440), which were consistent with the standard Fe_3_O_4_ diffraction data, further indicating that This further demonstrates that liposomes successfully cap Fe_3_O_4_ without changing the crystalline structure, and maintains the good properties of magnetic particles (Fig. 2F). The Raman spectra showed a G peak at 1570 cm^−1^ and a D peak at 1350 cm^−1^ with an IF/IG of 1.018, indicating that the surface of CD146-LMS contains a large number of hydroxyl and carboxyl groups (Fig. 2G). AFM, SEM, and TEM observations showed that CD146-LMS was spherical with different sizes, regular shape, no agglomeration, and size distribution between 100 and 600 nm (Fig. 2H-J).

### Cytotoxicity of CD146-LMS and growth of CD146 + BMSCs

**Fig. 3:**
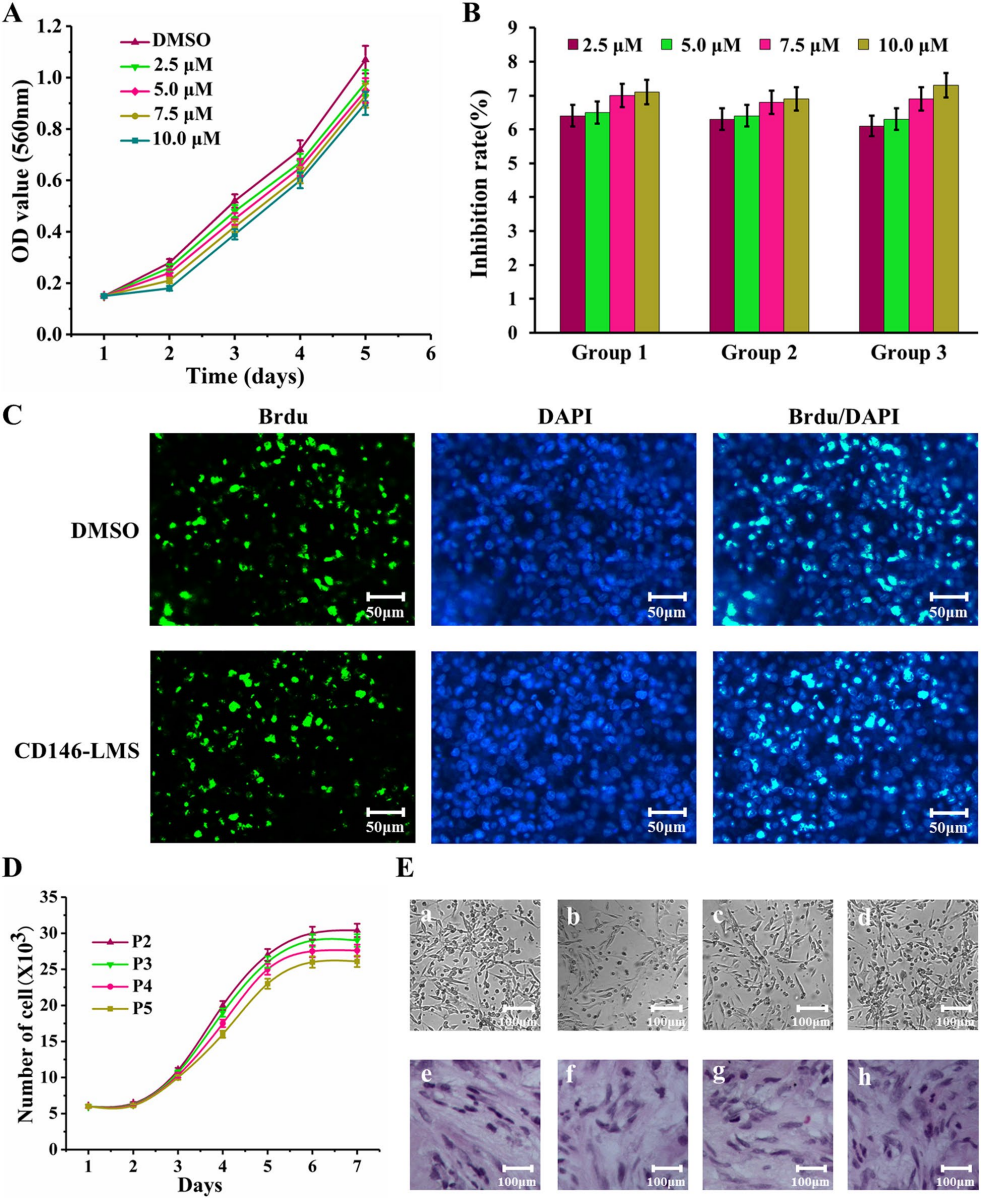
Investigate the impact of CD146-LMS on the cytotoxicity of BMSCs and the growth of CD146 + BMSCs. **A** The effect of CD146-LMS on BMSCs cell growth; (**B**) Inhibitory effect of different concentrations of CD146-LMS on BMSCs cells; (**C**) BrdU staining for cell proliferation; (**D**) The growth of CD146 + BMSCs cells; (**E**) Morphological observation of CD146 + BMSCs cells, which includes the growth morphology of CD146 + BMSCs cells of P2—P5 generations (a-d) and HE staining of CD146 + BMSCs cells of P2—P5 generations (e–h)

The addition of 2.5, 5, 7.5, and 10 μM CD146-LMS did not show significant cytotoxicity compared to the DMSO control group, and the cell inhibition rate was between 6 and 8% in all three experimental groups at day 5 of culture (Fig. 3A, B). The BrdU-positive cell rate was not significantly different from that of the DMSO-treated group after 10 μM CD146-LMS was added to the cells and cultured for 72 h (Fig. 3C). The growth curves of the BMSCs cells showed that the P2—P5 generation cells grew slowly in the first 2 days, grew the fastest from 3–6 days, and reached the optimal state after 6 days (Fig. 3D). The morphology of the CD146 + BMSCs cells was a long shuttle shape. There was no obvious difference in the shape of the P2—P5 generation cells, and HE staining showed that the cytoplasm of the P2—P5 generation CD146 + BMSCs cells was light blue, and the arrangement had a certain direction (Fig. 3E).

### Cell surface antigen detection

**Fig. 4:**
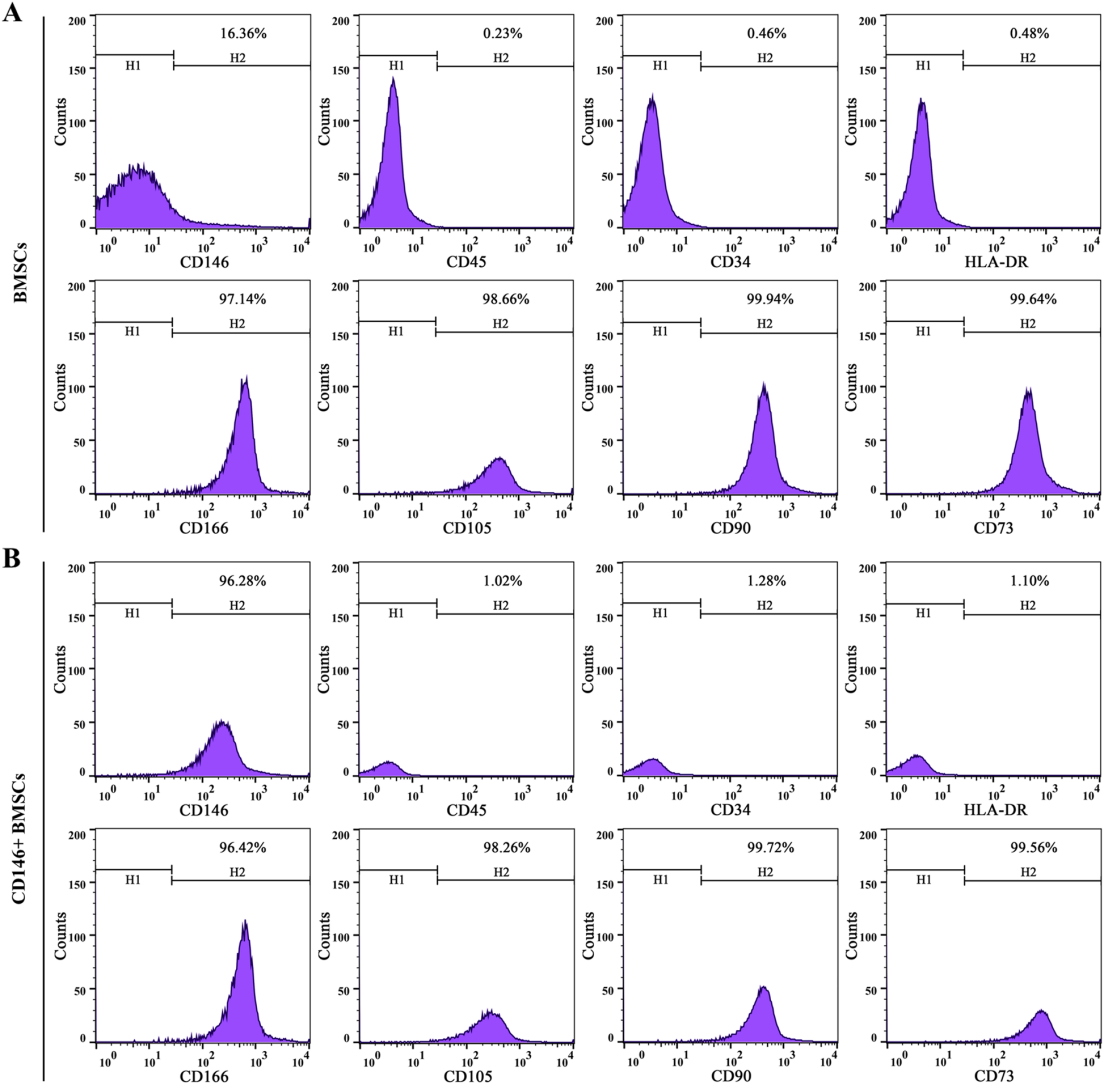
Cell surface antigen assay of P3 generation BMSCs. **A** Surface antigen detection of BMSCs; (**B**) Surface antigen detection of BMSCs CD146 + BMSCs

Flow results showed that both P3 generation BMSCs and CD146 + BMSCs cells highly expressed CD166, CD105, CD90, and CD73, and the expression rates were higher than 96%. Both P3 generation BMSCs and CD146 + BMSCs cells hardly expressed CD45, CD34, and HLA-DR, and the expression rates were all lower than 2%, which was similar to the MSC surface antigen expression profile was similar. However, P3 generation BMSCs cells expressed CD146 at a low rate of 16.36%, and P3 generation CD146 + BMSCs cells expressed CD146 at a high rate of 96.28% (Fig. 4A, B).

### Detection of chondrogenic characteristics

**Fig. 5:**
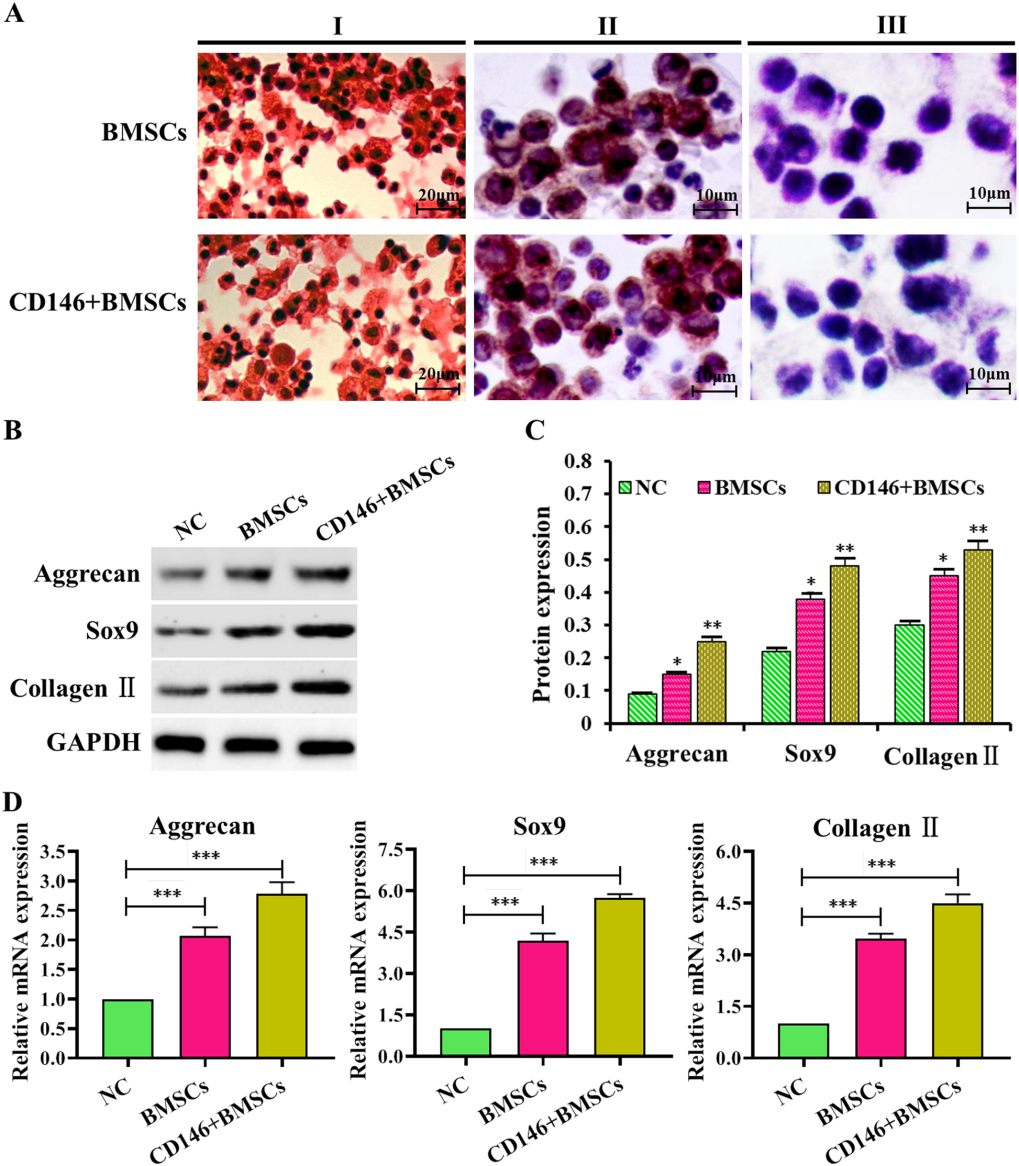
Induced chondrogenic properties assay. **A** I- III Alizarin red staining, type II collagen staining, and AB-PSA staining to assess chondrogenic properties, respectively; (**B**) Western blot to detect the protein expression of Aggrecan, Sox9, and Collagen II; (**C**) Relative expression of Aggrecan, Sox9, and Collagen II proteins; (**D**) RT-PCR to detect the relative expression of Aggrecan, Sox9, and Collagen II mRNA

The chondrogenic effect of cultured BMSCs and CD146 + BMSCs cells was evident with positive alizarin red staining, and calcium salts deposited by the forming osteoblasts were stained red. Type II collagen staining was positive, and the cell surface and intercellular components were stained tan. AB-PSA staining was positive, the nuclei were stained dark blue. The cell membranes were stained purplish-red. It was shown that both induced BMSCs and CD146 + BMSCs had the relevant phenotypes of normal chondrocytes (Fig. 5A). Aggrecan is widely distributed in the cartilage matrix, Sox9 is an initiator gene for chondrogenic differentiation, and Collage II is a major component of the cartilage extracellular matrix. To further demonstrate that BMSCs and CD146 + BMSCs underwent differentiation toward chondrocytes after induction, BMSCs and CD146 + BMSCs were detected to express characteristic products of chondrocytes at protein and molecular levels, respectively. The results of the Western blot assay showed that the protein expression of Aggrecan, Sox9, and Collage II was significantly elevated in the BMSCs group compared with the NC group (*P* < 0.05), and the elevated protein expression of Aggrecan, Sox9 and Collage II in the CD146 + BMSCs group and its significant (*P* < 0.01) (Fig. 5B, C). RT-PCR assay results showed that compared with the NC group, the Aggrecan, Sox9, and Collage II protein expression in both the BMSCs group and CD146 + BMSCs group were elevated and its significant (*P* < 0.001) (Fig. 5D). All these results indicated that BMSCs and CD146 + BMSCs were successfully induced to differentiate into chondrocytes.

### Cartilage construction and histological examination

**Fig. 6:**
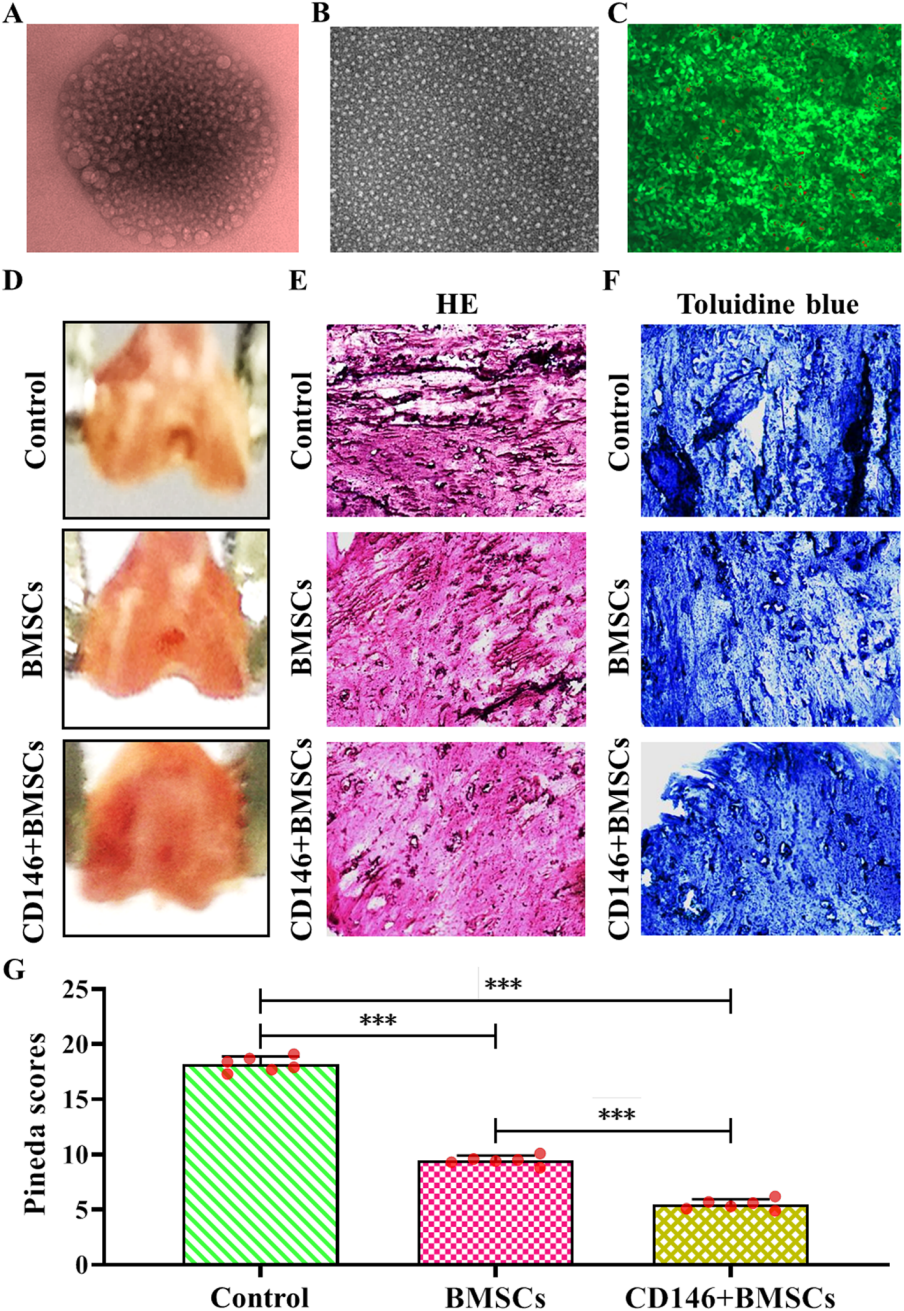
Cartilage construction and histological results. **A** Sodium alginate microspheres were observed under the inverted microscope; (**B**) Sodium alginate microspheres were observed under a laser confocal microscope. (**C**) Sodium alginate microspheres were observed under a fluorescence microscope. (**D**) Repair effect map of defective cartilage in mice; (**E**) HE staining of repaired cartilage tissue (× 200); (**F**) Toluidine blue staining of repaired cartilage tissue (× 200); (**G**) Histological score of repaired cartilage

Cells were distributed in the cavity formed by sodium alginate microspheres by inverted microscopy (Fig. 6A), and cells were seen to be spherical in shape by confocal microscopy (Fig. 6B). Fluorescence microscopy showed that most of the cells survived and emitted green fluorescence; a small number of cells died and emitted red fluorescence (Fig. 6C). The repair results of the defective cartilage in mice showed (Fig. 6D) that the control group had a very obvious depression, with a clear boundary outline with the surrounding normal cartilage. The BMSCs group had an obvious depression but was tightly connected with the surrounding normal cartilage, with a relatively flat surface, and the boundary outline was unclear. The CD146 + BMSCs group had an inconspicuous depression, with a flat surface of the cartilage tightly connected with the surrounding normal cartilage, and the boundary outline largely disappeared. The results of HE and toluidine blue staining of the tissues showed (Fig. 6E-F, and Figure S1A, B) that there was a small amount of fibrous tissue in the cartilage tissues of the control group, with some degeneration of the surrounding cartilage, and no heterochromia was seen in the matrix staining, with obvious local depression. The cartilage surface in the BMSCs group was flatter and well connected to the surrounding bone. The cartilage surface was flatter in the CD146 + BMSCs group, with normal morphology and structure of the newborn subchondral bone, better connection with the surrounding bone, and continuous and flat cartilage surface. The scoring results showed (Fig. 6G) that compared with the control group, the scores of the BMSCs group and CD146 + BMSCs were significantly lower. The scores of the CD146 + BMSCs group were significantly lower than those of the BMSCs group, and the difference was statistically significant in all cases (*P* < 0.001).

## Discussion

Repair of articular cartilage damage is a major clinical challenge, and although there are a variety of methods for treating cartilage damage, none of their long-term effects are ideal [8]. Currently, commonly used treatments include autologous chondrocyte transplantation, osteochondral grafting, and microfracture techniques. Although autologous chondrocyte transplantation has shown good results in prospective randomized trials, there are some problems, including the need for two surgeries and high treatment costs [33]. Osteochondral grafting has favorable short-term results, but the failure rate of osteochondral grafting is significantly higher at 10 years after surgery compared with autologous chondrocyte grafting [34]. Microfractures have better short-term symptomatic improvement, but fibrous tissue regeneration is seen at 2 years postoperatively and there is a recurrence of clinical symptoms [35]. Currently, the newest and hottest research strategy for cartilage injury repair is cartilage tissue engineering based on cell therapy, i.e., seed cells combined with bioscaffolding materials and prochondrogenic factors are implanted into the damaged area in vivo for cartilage injury repair [5, 36, 37]. Seed cell selection is the foundation and prerequisite of cartilage tissue engineering and also the key [38]. BMSCs have stem cell properties that can differentiate into bone, cartilage, adipose, and other cells and are a popular choice of seed cells for cartilage tissue engineering [39–41]. Studies have shown that MSCs are a heterogeneous population with multiple subpopulations, in which the potential biological properties of different subpopulations of stem cells are not well understood [38, 42]. CD146, an adhesion molecule mainly expressed in endothelial cells, has been identified as an early MSCs marker [43]. Studies have shown that CD146 might be a new cell surface marker for cartilage progenitor cell population in the late-stage osteoarthritis [25]. CD146 + cells have greater potency than CD146- cells for cartilage protection and can suppress Th17 cell activation [24]. Downregulation of CD146 accelerates cells senescence in human umbilical cord blood-derived MSCs [44]. Endometrial mesenchymal stem/stromal cells, and even more, the CD146 + subpopulation, possess exosomal secretomes with strong immunomodulatory miRNA attributes [45]. CD146 + perivascular cells, a component of MSCs, play a key role in the wound healing process [46]. CD146 + cells isolated from human fat aspirates show immunomodulatory effects during bone formation [47]. CD146 + MSC show greater migratory potential to repopulate intervertebral discs [48]. However, the role of CD146-positive BMSCs cell subsets as seed cell types in cartilage defect repair is unclear. Therefore, it is important to use CD146 + BMSCs subpopulation in BMSCs cells for cartilage tissue repair by sorting and culturing them.

This study used immunomagnetic bead sorting to sort CD146-positive subpopulations from bone marrow mesenchymal stem cells. Currently, immunomagnetic beads and flow cytometry are the most common sorting techniques. Among them, when flow cytometry sorts a large number of cells, the purity and activity of the sorted cells are not high due to the long time required. Immunomagnetic bead sorting technology can guarantee more than 80% positive rate, which is internationally recognized in the application of tissue engineering cell sorting, and at the same time, it has the advantages of less harm to cells, easy operation, shorter time, better sensitivity and selectivity of the sorting process, and is able to sort out cells with high purity and activity, which is more conducive to the later experimental research [49–51]. In this study, we independently developed a CD146-LMS with the function of CD146 target recognition; CD146-LMS has a small particle size, which is helpful for the homogeneous and stable dispersion in solution.CD146-LMS is positively charged, and the microspheres charged on the surface have better dispersion due to the electrostatic repulsion between each other, which is helpful for dispersion of the microspheres in hydrophilic solution. The positively charged LMS favors binding to negatively charged cells. In addition, CD146-LMS has better magneticm and lower cytotoxicity, and the positive expression rate of sorted CD146 + BMSCs was 96.28%, which is capable of specifically, safely and efficiently sorting out the subpopulation of CD146 + BMSCs and expanding and culturing them, and is able to satisfy the application of chondrogenic induced differentiation for cartilage damage repair.

For tissue-engineered cartilage to be used in clinical applications, scaffolding materials must be involved to enable three-dimensional culture and observation of cells [52, 53]. Sodium alginate has been widely used in cartilage tissue engineering research [54, 55]. Studies have shown that mesenchymal stem cells can survive and proliferate well within sodium alginate microspheres and can form cartilage [56]. In this study, we utilized microspheres formed by the interaction between sodium alginate solution and CaCl_2_ solution as three-dimensional scaffolding materials to wrap CD146 + BMSCs and stereocultured CD146 + BMSCs in vitro. The results showed that CD146 + BMSCs had good compatibility with sodium alginate microspheres, in which they could survive well, maintain their spherical shape, and tend to aggregate gradually. In addition, it was observed that a small number of CD146 + BMSCs would die after the cells were complexed with sodium alginate, which was consistent with the literature report and was considered to be caused by osmotic pressure changes [57]. Due to the small amount of cell death, it would not affect the results of the experiment. In order to exclude the effect of host immune factors on the grafts, this study used the knee joint of nude mice to make an articular cartilage injury model, utilizing BMSCs and CD146 + BMSCs as seed cells and sodium alginate calcium gel as scaffolding material. Sodium alginate microspheres encapsulating CD146 + BMSCs were introduced into cartilage defects in mice for repair studies, and it was found that a subpopulation of CD146 + BMSCs better promoted the repair of articular cartilage damage. It is suggested that the specific subtype of BMSCs will be a new direction for tissue engineering research, which provides a new idea for selecting seed cells for tissue engineering and lays the foundation for stem cell clinical treatment and research application. In this study, only one-time point of cartilage repair was observed, and cartilage damage repair is a dynamic and gradual change process, therefore, it is necessary to improve the relevant experiments further and set up multiple time points to observe cartilage repair dynamically. In addition, although this study confirmed that CD146 + BMSCs can better promote cartilage repair, its specific mechanism is unclear, and further studies are needed to clarify its mechanism.

## Conclusion

In conclusion, the CD146-LMS developed independently in this study successfully sorted out CD146-positive subpopulation cells from BMSCs, which had good proliferation characteristics and good survival status. CD146 + BMSCs are a good source of seed cells for cartilage tissue engineering. The CD146 + BMSCs subpopulation induced by in vitro chondrogenesis in combination with sodium alginate could maintain thechondrocyte phenotype in the damaged joints of nude mice, and Could maintain the chondrocyte phenotype and better promote the repair of articular cartilage damage.

### Supplementary Information

**Supplementary Material 1.**

## Data Availability

The analyzed data sets generated during the study are available from the corresponding author on reasonable request.
