# Supplementary material for: Specific lipid magnetic sphere sorted CD146-positive bone marrow mesenchymal stem cells can better promote articular cartilage damage repair
Source: BMC Musculoskelet Disord. 2024 Apr 1;25:253. doi: 10.1186/s12891-024-07381-6 (PMC10983655; doi:10.1186/s12891-024-07381-6)
Supplement: Supplementary file 1 — Supplementary Material 1. [file 12891_2024_7381_MOESM1_ESM.docx]

**Supplementary information**

**
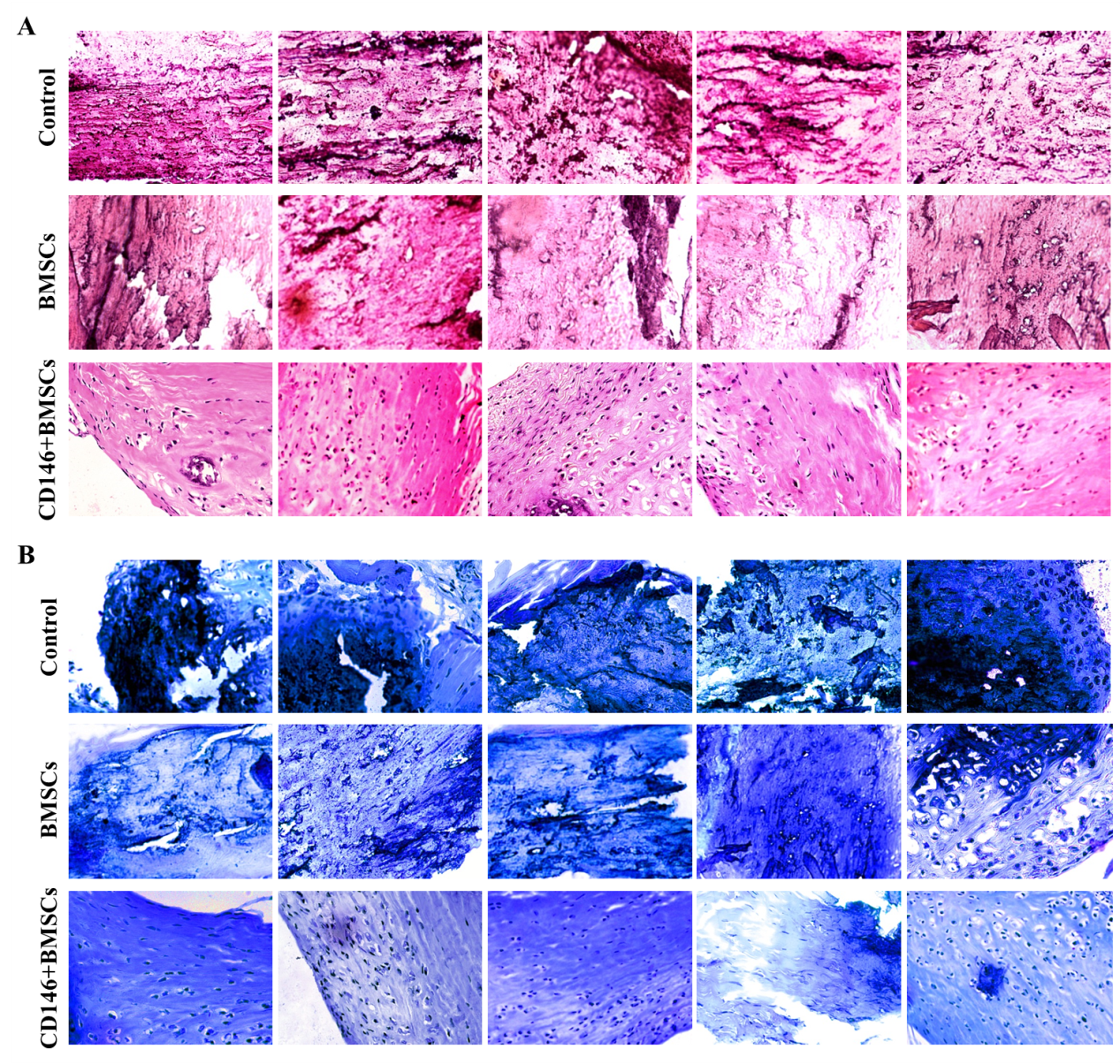
**

**Figure S1.** Tissue HE staining and toluidine blue staining. A. HE staining of repaired cartilage tissue (×200); B. Toluidine blue staining of repaired cartilage tissue (×200)

**Raw data for Figure 5B**


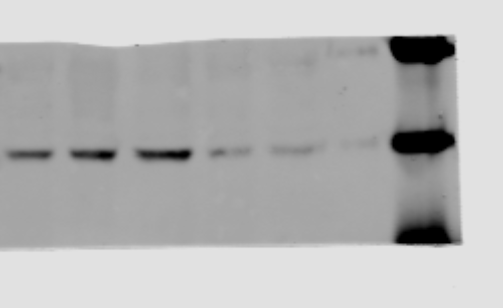


Aggrecan


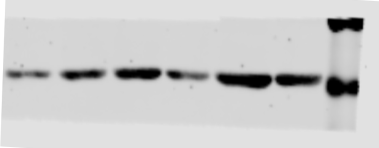


Sox9


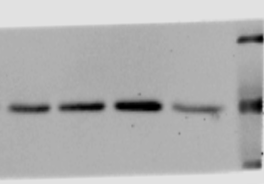


Collagen Ⅱ


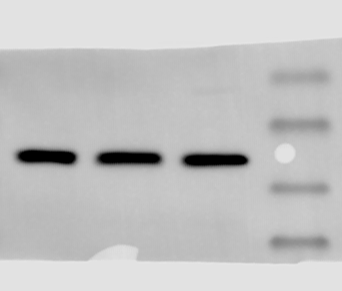


GAPDH
